# The new world of RNA diagnostics and therapeutics

**DOI:** 10.1186/s13046-023-02752-8

**Published:** 2023-07-29

**Authors:** Giovanni Blandino, Roberto Dinami, Marco Marcia, Eleni Anastasiadou, Brid M. Ryan, Alina Catalina Palcau, Luigi Fattore, Giulia Regazzo, Rosanna Sestito, Rossella Loria, Ana Belén Díaz Méndez, Maria Chiara Cappelletto, Claudio Pulito, Laura Monteonofrio, George A. Calin, Gabriella Sozzi, Jit Kong Cheong, Ranit Aharonov, Gennaro Ciliberto

**Affiliations:** 1 Translational Oncology Research Unit, IRCCS, Regina Elena National Cancer Institute, Rome, Italy; 2 EMBL, Grenoble, France; 3 Department of Clinical and Molecular Medicine, Sapienza University, Rome, Italy; 4 MiRNA Therapeutics, London, UK; 5 SAFU Laboratory, IRCCS Regina Elena National Cancer Institute, Rome, Italy; 6 Preclinical models and new therapeutic agents Unit, IRCCS Regina Elena National Cancer Institute, Rome, Italy; 7 Unit of Cellular Networks and Molecular Therapeutic Targets, IRCCS Regina Elena National Cancer Institute, Rome, Italy; 8 MDAnderson, University of Texas, Houston, USA; 9 IRCCS, National Cancer Institute, Milan, Italy; 10 National University of Singapore Yong Loo Lin School of Medicine, NUS Centre for Cancer Research and Mirxes Lab Pte Ltd, Singapore, Singapore; 11 Pangaea Biomed, Tel Aviv, Israel; 12 Scientific Direction, IRCCS Regina Elena National Cancer Institute, Rome, Italy

**Keywords:** RNA structure, Cell cycle regulation, RNA evolution, Epigenetics, Splicing

## Abstract

The 5th Workshop IRE on Translational Oncology was held in Rome (Italy) on 27–28 March at the IRCCS Regina Elena National Cancer Institute. This meeting entitled “The New World of RNA diagnostics and therapeutics” highlightes the significant progress in the RNA field made over the last years. Research moved from pure discovery towards the development of diagnostic biomarkers or RNA-base targeted therapies seeking validation in several clinical trials. Non-coding RNAs in particular have been the focus of this workshop due to their unique properties that make them attractive tools for the diagnosis and therapy of cancer.

This report collected the presentations of many scientists from different institutions that discussed recent oncology research providing an excellent overview and representative examples for each possible application of RNA as biomarker, for therapy or to increase the number of patients that can benefit from precision oncology treatment.

In particular, the meeting specifically emphasized two key features of RNA applications: RNA diagnostic (Blandino, Palcau, Sestito, Díaz Méndez, Cappelletto, Pulito, Monteonofrio, Calin, Sozzi, Cheong) and RNA therapeutics (Dinami, Marcia, Anastasiadou, Ryan, Fattore, Regazzo, Loria, Aharonov).

## Exploring miRNAs/mRNAs network in breast cancer progression

**Fig. 1:**
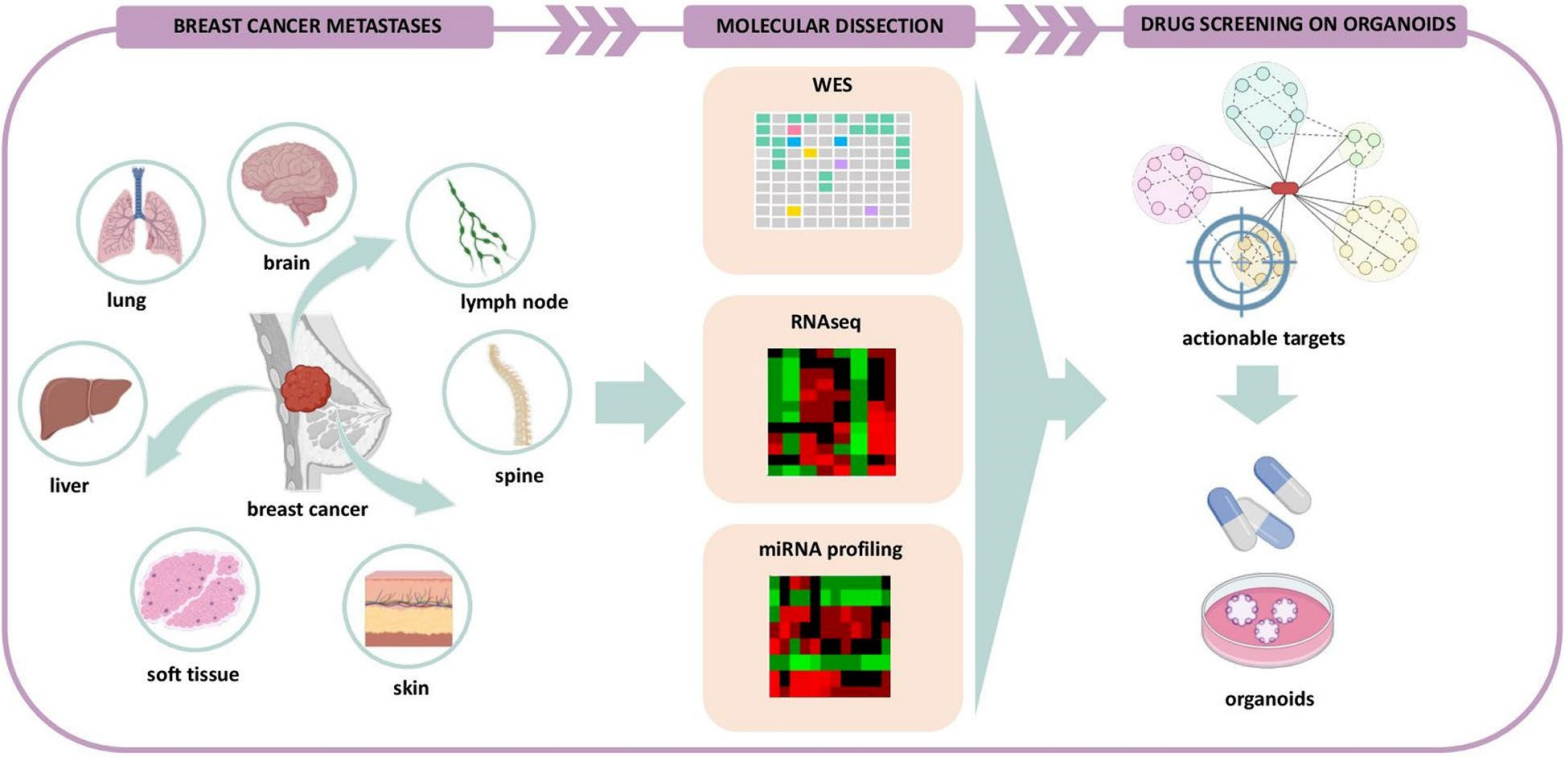
Molecular dissection of breast cancer metastases. We collected breast cancer metastases from different sites, and we performed multiomic analyses, including whole exome sequencing (WES), bulk RNA sequencing (RNAseq), and miRNA profiling. The integrated analyses allowed to the identification of altered pathways in breast cancer metastases and novel therapeutic approaches that will be evaluated on patients-derived organoids

*Giovanni Blandino* (IRCCS, Regina Elena National Cancer Institute, Rome, IT). Metastasis is the leading cause of cancer related mortality. Metastasis is an evolutionarily dynamic and multifaced process along which tumor cells gain the ability to escape from the primary site and colonize distant organs. Unlike primary tumors, metastatic patients have not strikingly benefited from therapeutic advances. A major obstacle to designing precise treatment for metastatic disease relies on the lack of mechanistic details of the dissemination process. Growing evidence has reported that the mutational landscape of metastatic lesions resembles largely that of the originating primary tumors thereby limiting the use of precision drugs directed to specific druggable gene mutations. Altogether these findings suggest that epigenetic alterations and aberrant expression of RNA on both coding and non-coding components might endow unique information to decipher molecularly metastasis. To fulfill this specific aim, we combined bulk-RNA seq data with microRNA profiling of a given set of breast cancer mets to identify miRNA/mRNA networks with pro-metastatic activities (Fig. 1). To functionally assess the contribution of the identified network we are actively pursuing the generation of organoids derived from fresh breast cancer-derived metastatic tissue lesions (Fig. 1). We have recently shown that organoids derived from breast cancer metastatic lesions carrying PIK3CA mutations can be efficaciously treated with PI3K-a inhibitor, Alpelisib irrespective of the metastatic site [1, 2]. This unique 3D-preclinical platform has proven to mostly recapitulate the genomic alterations and gene expression patterns of the originating metastatic tissue; thereby providing a robust cancer surrogate to test the impact of miRNA/mRNA networks in the metastatization process and unveil new metastatic targets to be tacklen therapeutically.

## Targeting TRF2 by LNPs-miR-182-3p in triple-negative breast cancer

*Roberto Dinami* (IRCCS, Regina Elena National Cancer Institute, Rome, IT). Telomeric repeat binding factor 2 (TRF2) is a member of the Shelterin complex involved in chromosome ends protection and telomere maintenance by T-loop formation [3, 4]. It is over-expressed in different cancer types and contributes to cancer progression [5, 6]. Here, we developed a miRNA-based approach to reduce TRF2 expression in vivo.

**Fig. 2:**
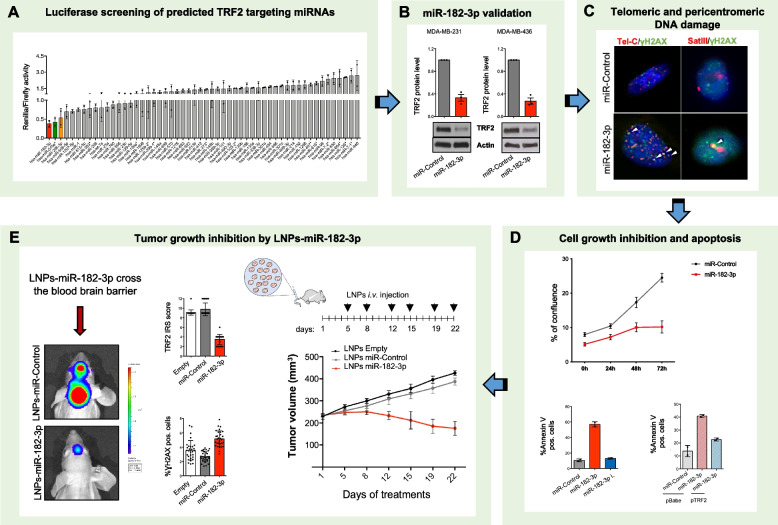
Targeting TRF2 by LNPs-miR-182-3p in triple-negative breast cancer.** A** Targeting of 3'UTR-TRF2 by selected miRNAs was tested by high-throughput luciferase screening. **B** Western blot of TRF2 expression in tumor cells transfected with miR-182-3p or miR-Control is shown. **C** Representative images show the telomeric and pericentromeric DNA damage induced by miR-182-3p in MDA-MB-231 cells. **D** Cell confluence or apoptosis activation in MDA-MB-436 cells, transfected with the indicated miRNAs, were monitored by live-cell imaging (Incucyte) or analysed by flow cytometry (FACS), respectively. **E** Scheduling of treatment (top/right panel); Analysis of tumor inhibition after treatment with LNPs-miR-182-3p or its relative controls (bottom/right panel); TRF2 and γH2AX expression in tumor tissues was analyzed by immunohistochemistry (IHC) (middle panel); Representative images show mice with artificial brain tumor generated by intracranial injection of MDA-MB-231. Mice were treated with LNPs-miR-182-3p or its control (left panel)

By performing a high-throughput luciferase screening, we identified miR-182-3p as a specific and efficient regulator of TRF2 able to induce DNA damage at telomeric and pericentromeric sites, eventually leading to strong apoptosis activation (Fig. 2A-D).

Interestingly, treatment with miR-182-3p-containing lipid nanoparticles (LNPs) induced tumor growth inhibition in advanced TNBC models including artificial brain metastasis (Fig. 2E). Our approach represents a possible therapeutic option for TNBC and its metastatic brain lesions.

## Diversity and functional implications of long non-coding RNA structures

**Fig. 3:**
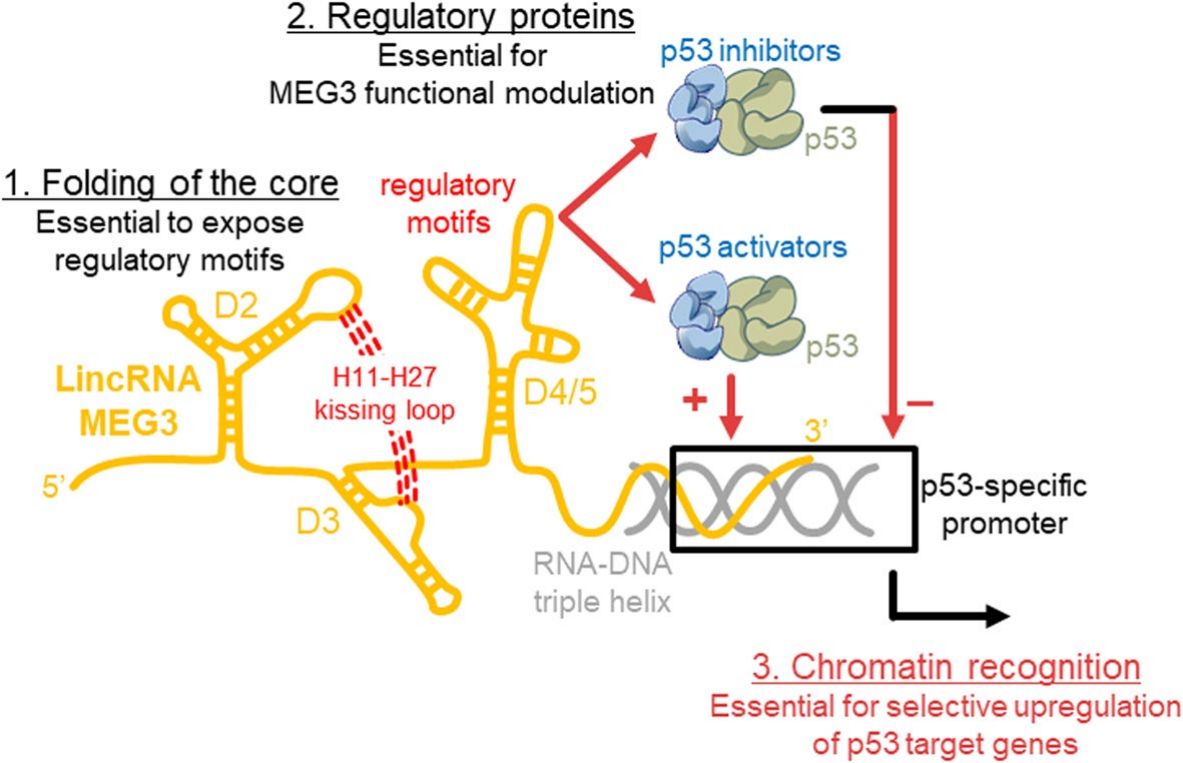
Mechanistic hypothesis for the p53-stimulatory role of MEG3. Folding of the MEG3 core exposes regulatory motifs to p53 activators/repressors to recruit p53 on chromatin, and fine-tune the expression of p53 target genes

*Marco Marcia* (EMBL, Grenoble, FR). Long non-coding RNAs (lncRNAs) are key regulators of gene expression, playing active roles in epigenetics, transcriptional and translational regulation, and chromatin scaffolding. However, because of their recent discovery and molecular complexity, lncRNAs are still very poorly characterized from a mechanistic perspective raising outstanding biological questions on how they selectively and efficiently tune gene expression. Our work describes the mechanistic complexity of lncRNAs from an interdisciplinary evolutionary, cellular, and structural perspective. We have specifically dissected the structural and functional properties of a human alternatively spliced lncRNA, called MEG3 which stimulates the p53 pathway preventing tumorigenesis. This lncRNA adopts a well-defined structural core, dictated by tertiary interactions that are essential for p53 stimulation and for inducing cell cycle arrest. Remarkably, we could visualize its 3D structure for the first time by AFM and SAXS at ~ 15 Å resolution. More recently, we have identified specific protein regulators of our target lncRNA. Some of these proteins are p53 activators responsible for its basal p53-stimulatory activity, while others are p53 repressors that inhibit its tumor-suppressing function (Fig. 3). Our work connects the 3D structure and protein interacting network of lncRNAs to their biological function and opens still-unexplored research perspectives to understand lncRNA biology at high resolution for exploiting their translational potential in RNA-based therapies.

## MiRNA-aided immunotherapies for Epstein-Barr virus (EBV)-associated lymphomas

**Fig. 4:**
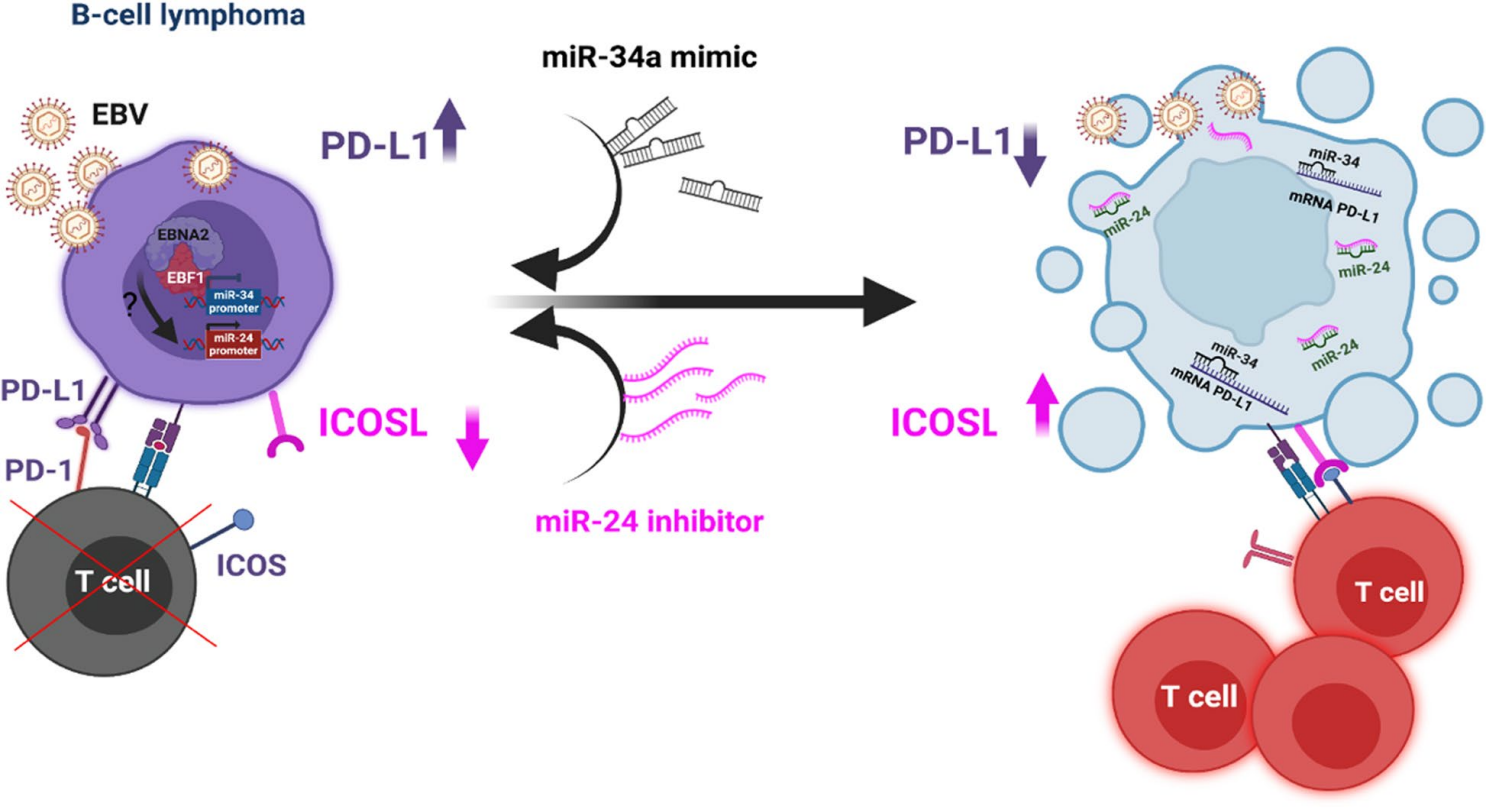
miRNA-aided therapy. Delivery of miR-34a mimics or miR-24 inhibitors miRNAs in B-cell lymphoma to fine-tune co-inhibitory and co-stimulatory signals, to facilitate immune escape (Biorender)

*Eleni Anastasiadou* (Sapienza University, Rome, IT) EBV belongs to the herpesvirus family and contributes to more than 200.000 cases of cancers per year globally, including Burkitt lymphoma, nasopharyngeal carcinoma, gastric cancer, and diffuse large B-cell lymphoma. The virus escapes T cell recognition and exerts its oncogenic potential using its latency programs. In particular, the presentation focused on the molecular mechanisms underlying viral immune escape through its major transforming protein, namely, Epstein-Barr nuclear antigen 2 (EBNA2). This viral protein induces the programmed death ligand-1 (PD-L1) immune checkpoint (IC) expression in DLBCL cell lines by downregulating PD-L1 targeting miR-34a [7]. We have recently shown how EBNA2 recruits early B-cell transcription factor 1 (EBF1) on the miR-34a promoter to silence its expression. Further, by using 3D microfluidic models, [7] we demonstrated for the first time that overexpression of miR-34a promotes T cell infiltration and killing of EBNA2 expressing DLBCL cells. These results paved the way for an international patent on RNA-aided immunotherapies (No.: WO2019232160). Using this model system, it was further shown that, the combination of miR-34a and anti-PD-L1 antibodies was more efficient in increasing the tumor immunogenicity than either anti-PDL1 or miR-34a alone. Subsequently, we illustrated how the same EBV-encoded protein negatively affects the inducible T cell costimulator (ICOSL). EBNA2 reduces ICOSL by upregulating miR-24 (unpublished data). Thus, EBNA2 seems to compromise tumor immunogenicity by simultaneously increasing PD-L1 by downregulating miR-34a and reducing ICOSL expression by increasing miR-24 (Fig. 4). Since cancer immunotherapies based on only anti-PD-L1 antibodies have shown low overall response rate (ORR), we propose a novel combinatorial miRNA-aided immunotherapy approach in which, IC targeting miRNAs in combination with antibodies may prove useful and provide a better outcome of immunotherapies [8].

## Applications of RNA activation in oncology

*Bríd M. Ryan* (MiRNA Therapeutics, London, UK). Small activating RNAs (saRNAs) are short double-stranded oligonucleotides that activate transcription within the nucleus through an evolutionarily conserved mechanism. MiNA Therapeutics has successfully designed saRNAs against a wide range of drug targets, including transcription factors, cytokines, receptors, and intracellular proteins. The translational read across of saRNA, therefore, extends from haematology to autoimmune diseases, metabolic diseases, and oncology, among others. MTL-CEBPA is a first-in-class saRNA therapy [9, 10]. Its mechanism of action involves upregulation of the transcription factor C/EBP-α (CCAAT/enhancer-binding protein alpha), which is a master regulator of the myeloid cell lineage. Decreased C/EBP-α expression frequently occurs in the context of cancer, with loss of C/EBP-α leading to a block in myeloid differentiation and an accumulation of MDSCs [11]. The saRNA is packaged within myeloid-targeting lipid nanoparticles and, when delivered to myeloid cells, restores C/EBP-α protein to normal levels to drive differentiation of immature myeloid cells towards a less immunosuppressive phenotype. Overcoming immunosuppression in the TME is a key step in the cancer immunity cycle (CIC). MDSCs are linked with both primary and secondary checkpoint blockade resistance [12] and represent an active target for improving ICB clinical efficacy [13]. In preclinical studies, combination treatment with MTL-CEBPA and anti-PD-1 showed marked synergistic abrogation of tumour progression [14]. Further, we found that MTL-CEBPA abrogates the immune suppressive activity of macrophages in syngeneic mouse models and demonstrated that the anti-tumour activity was mediated by CD8 + T cells [9]. TIMEPOINT is a phase 1A/B, first-in-human, open-label, multicenter study (NCT-04105335) that evaluated the safety, tolerability, pharmacokinetics, and efficacy of MTL-CEBPA in combination with pembrolizumab in 50 adult patients with advanced solid tumours. The drug combination is well tolerated and has an overall manageable toxicity profile. Clinical activity was observed with an overall disease control rate of 35% and 4 confirmed responses in mesothelioma, ovarian cancer, atypical lung neuroendocrine tumour, and intrahepatic cholangiocarcinoma. Across all tumours, multimodal analyses of paired patient biopsies pre- and post-treatment demonstrated that combined MTL-CEBPA and pembrolizumab treatment resulted in significantly increased intratumoural infiltration of proliferating cytotoxic CD8 + T cells and inflammatory HLA-DR + myeloid populations, as well as positive enrichment of associated immune activation gene pathways and reduction in genes associated with immunosuppression. Among immune-desert tumours, the combination of MTL-CEBPA with pembrolizumab transforms the TME into an immune-inflamed phenotype, as evidenced by the increased T cell chemokines and cytotoxic T cells. Among immune-inflamed tumours, the combination treatment led to a reduction in PMN-MDSCs in the tumour and circulation. Collectively, these data suggest a positive immunomodulatory TME effect of the combination of MTL-CEBPA with pembrolizumab. Through a wider lens, addressing each point of the cancer immunity cycle is central to achieving complete and durable cancer remission for patients. Using the combination of myeloid-targeted cell delivery with small activating RNAs, MiNA has developed a platform that can address multiple nodes of the CIC that have not been vulnerable to targeting with previous drug classes.

## CircRNAs and breast cancer metabolism

**Fig. 5:**
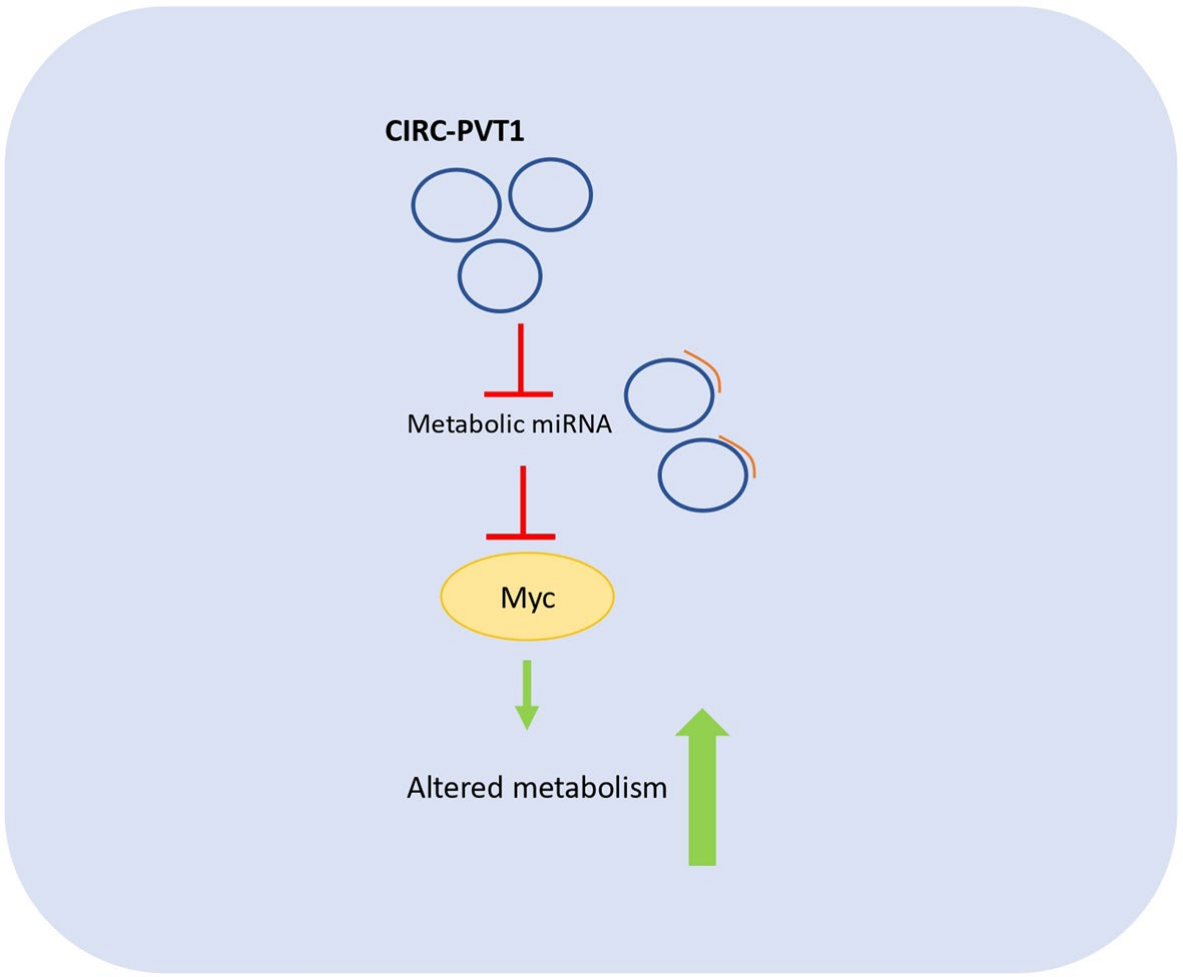
CircPVT1 exerts an oncogenic activity by acting as a miRNAs sponge regulating the expression of pivotal metabolic genes

*Alina Catalina Palcau* (IRCCS, Regina Elena National Cancer Institute, Rome, IT). Triple-negative breast cancer is the most aggressive breast cancer subtype because it lacks molecular targets. In this context, we investigated the role of a peculiar circular RNA called circPVT1 which originates from the PVT1 gene that encodes also for a lncRNA. Moreover, the PVT1 locus is located only 54 kb downstream MYC locus thus not excluding a possible synergistic effect between circPVT1 and MYC [15, 16]. Intriguingly, among the oncogenic activities of circPVT1, the aberrant expression of this circRNA molecule in breast cancer contributes to metabolism alteration. We found that circPVT1, mainly localized in the cytoplasm, exerts its pro-tumorigenic activity through the activation of a transcriptional axis controlling the expression of pivotal enzymes involved in specific metabolic pathways (Fig. 5).

## The multifaceted role of miR-579-3p in melanoma: diagnostic and therapeutic implications

**Fig. 6:**
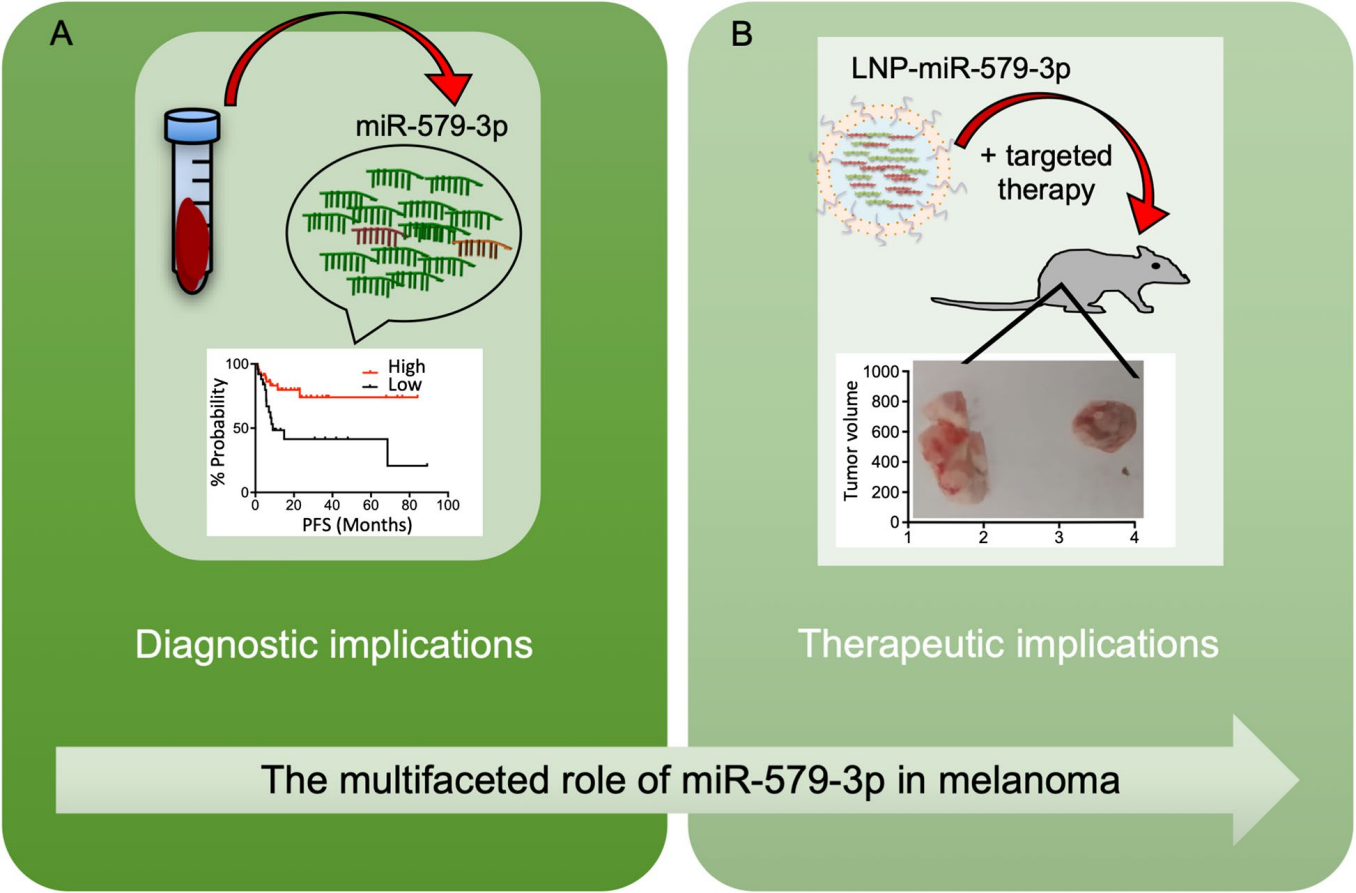
The figure represents the multifaceted role of miR-579-3p in BRAF-mutant metastatic melanoma.** A** The circulating levels of miR-579-3p can be easily determined in liquid biopsies and are able to predict response to targeted therapy in patients. **B** Lipid nanoparticles (LNPs) delivering miR-579-3p can prolong the efficacy of targeted therapies in melanoma xenograft models

*Luigi Fattore* (IRCCS, Regina Elena National Cancer Institute, Rome, IT). The multifaceted role of miR-579-3p in melanoma has been focused on a novel oncosuppressive miRNA previously identified as a master regulator of resistance to targeted therapies in BRAF-mutated melanomas [17]. From here, the potential therapeutic and diagnostic implications of miR-579-3p have been investigated. Therapeutically, the systemic delivery of miR-579-3p through lipid nanoparticles (LNPs) impairs the development of drug resistance in vivo (unpublished results) because this miRNA can target the BRAF oncogene [17]. From the diagnostic standpoint, it has been demonstrated that the circulating levels of miR-579-3p are able to predict response to targeted therapy in patients [18]. Finally, Dr. Fattore has presented unpublished results on the molecular regulation of miR-579-3p in melanoma (Fig. 6).

## Different genome-wide approaches to identify microRNAs related to DLBCL resistance to immune-chemotherapy

**Fig. 7:**
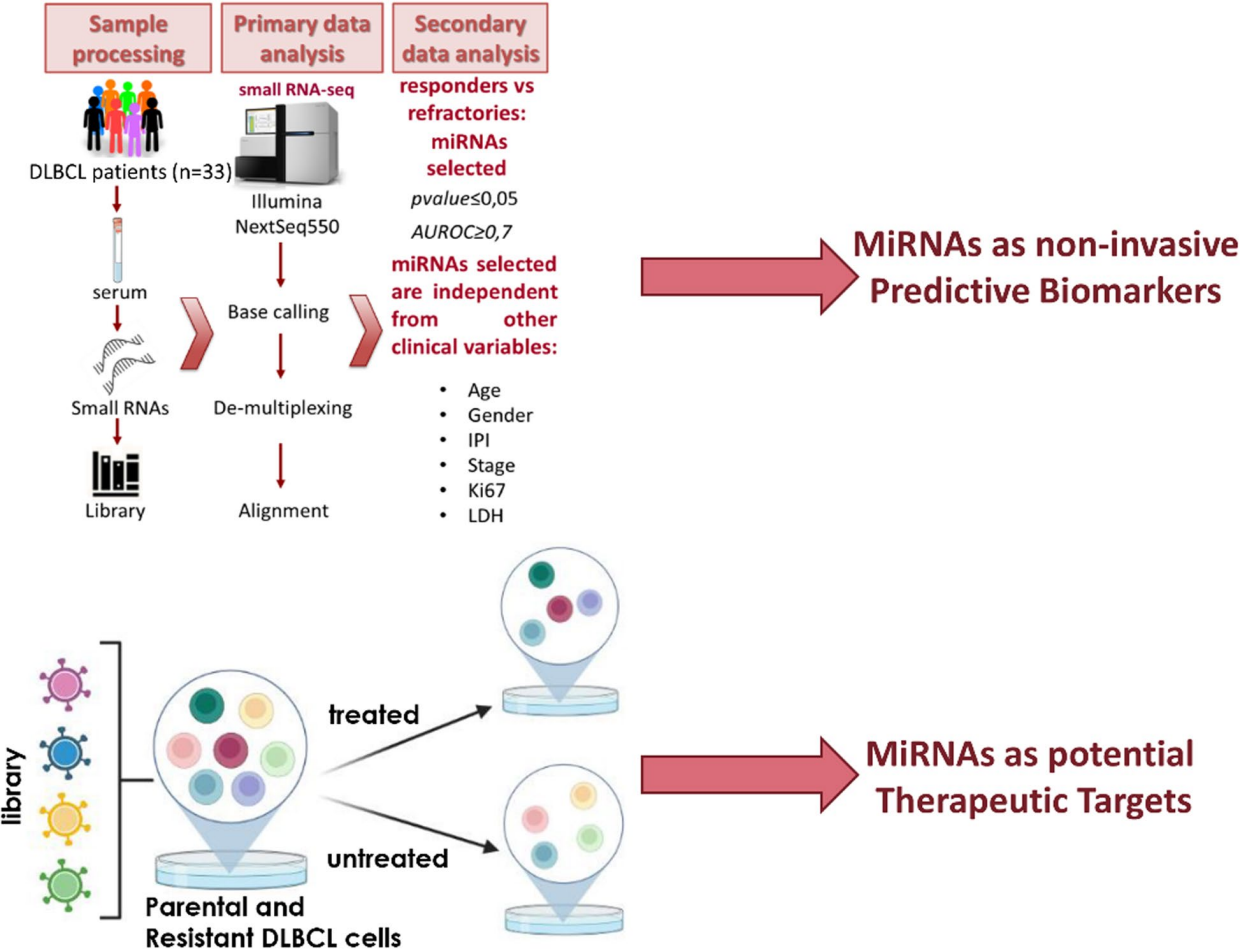
Genome-wide approaches to study miRNA involved in DLBCL response to treatment. (A) Small-RNA sequencing on serum samples from DLBCL patients responding or refractory to R-CHOP treatment to identify miRNAs as predictive biomarkers. (B) CRISPR/Cas-9 library screening on a cell model of drug resistance to identity miRNAs as potential therapeutic targets (image created with BioRender.com)

*Giulia Regazzo* (IRCCS, Regina Elena National Cancer Institute, Rome, IT). Diffuse large B-cell lymphoma (DLBCL) is a heterogeneous and aggressive cancer. Patients’ refractory to the first-line treatment still represent an unmet medical need [19]. MicroRNAs (miRNAs), non-coding RNA regulating gene expression, impact several cancer processes including drug resistance [20]. To delineate the role of miRNAs in DLBCL response to treatment we performed:

(i) A miRNA profiling, by small-RNA Seq, in serum samples of patients that led to the identification of miRNAs differentially expressed in R-CHOP refractory and responding subjects (Fig. 7A). Two of the identified miRNAs showed a functional involvement in DLBCL cell growth decreasing cell proliferation, viability and displaying, based on in-silico analyses, putative targets involved in DLBCL relevant pathways such as the Myc signaling.
(ii) A global loss-of-function screening on in-vitro models of drug resistance (Fig. 7B), using a miRNA-specific CRISPR-Cas9 library to study the miRNA role in response to treatment from a mechanistic point of view. This approach led to the identification of a plethora of miRNAs potentially involved in drug resistance. These results may delineate miRNAs with a notable impact on DLBCL response to treatment and possibly translate these findings into therapeutic interventions.

## Deciphering the role of endothelin-1/ZEB1/gasdermin E axis in the metastatic progression of high-grade serous ovarian cancer

**Fig. 8:**
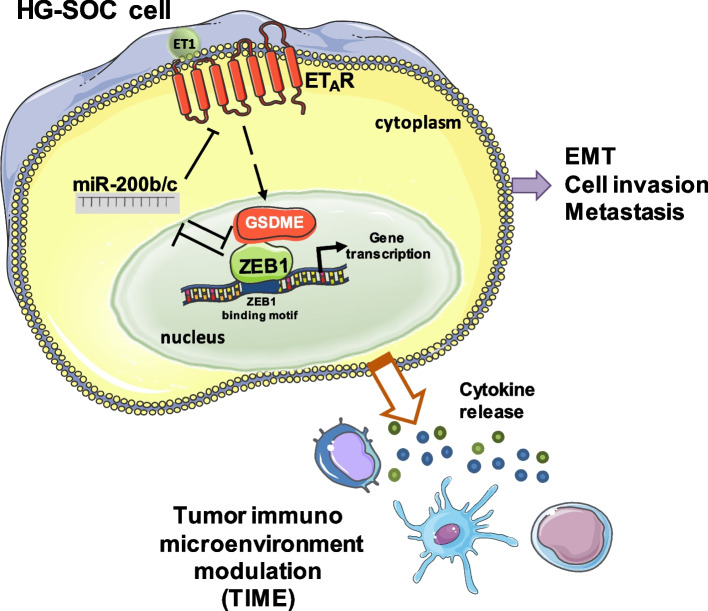
A schematic diagram illustrating the ability of ET-1-driven ET_A_R/miR-200/ZEB1/GSDME circuitry to fuel metastasis progression and reprogram the tumour immune microenvironment (TIME) in HG-SOC cells

*Rosanna Sestito* (IRCCS, Regina Elena National Cancer Institute, Rome, IT). High-grade serous ovarian carcinoma (HG-SOC) represents the most common and aggressive histotype of ovarian cancer. We identified a novel multi-factorial regulatory circuit controlled by endothelin-1 (ET-1) that integrates gasdermin E (GSDME), a key effector in inflammatory pyroptosis [21], with the endothelin A receptor (ET_A_R)/ZEB1 axis [22], by using patient-derived HG-SOC preclinical models. In this scenario, we pinpointed a critical role of miR-200b/c in curtailing GSDME abundance by targeting both ET_A_R and ZEB1 expression. Interestingly, the provided findings offer a hint into the non-pore forming function of GSDME, unravelling the ability of GSDME to act as a transcriptional cofactor of ZEB1 to fuel the ET-1-triggered metastatic progression and tumor immune microenvironment reprogramming. This work opens new perspectives of therapeutic strategies to balance the novel functions of GSDME by targeting ET-1 signaling to improve the poor prognosis of HG-SOC patients (Fig. 8).

## Exploring molecular traits and immunogenicity of osteosarcoma for new therapeutic approaches

**Fig. 9:**
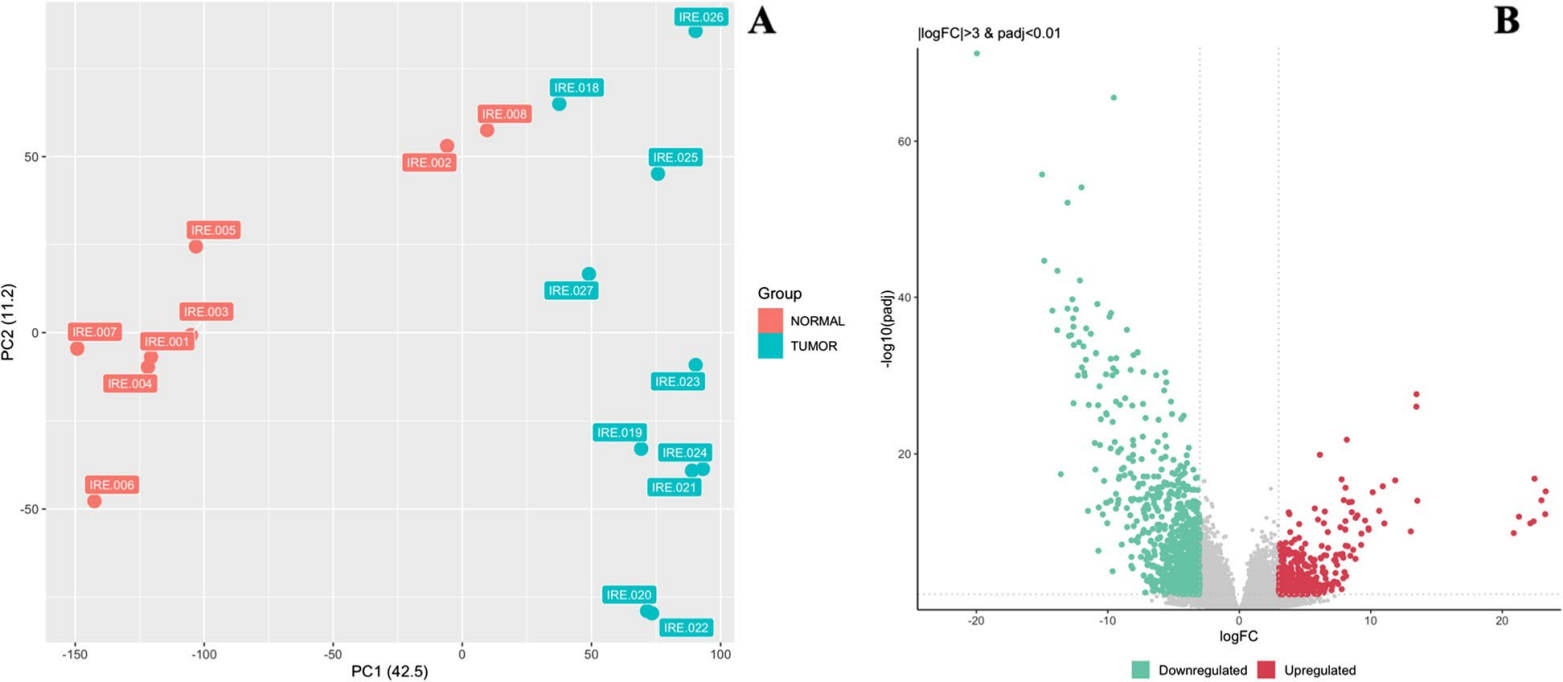
Panel **A** PCA plot. Panel **B** Volcano plot for OS vs normal bone specimens

*Rossella Loria* (IRCCS, Regina Elena National Cancer Institute, Rome, IT). Aiming to identify osteosarcoma (OS)-specific targetable antigens, we conceived a comparative transcriptomic analysis by sequencing the entire transcriptome of two distinct cohorts consisting of normal bone (cohort A) and OS (cohort B) specimens. At present, we have completed the bioinformatics analysis of 8 samples from cohort A and 10 of cohort B. The clustering of normal bone and OS samples by Principal component analysis (PCA) revealed significant variations between these two cohorts confirmed by differential gene expression analysis results (Fig. 9A and B). Based on the fold change (log3fold) differences between tumor and control samples, we obtained a large list of genes that are strongly differentially expressed among samples of the two cohorts, thus supporting the feasibility of this project. Next, we performed gene ontology analysis to better understand the potential impact of the signaling pathways on OS tumorigenesis and progression. In particular, we are specifically focusing on membrane cell surface molecules as potential therapeutic targets. We found that one of the most significant pathways probably involved in OS pathogenesis is related to the activity of the transmembrane tyrosine kinase receptors (GO:0004714), especially ephrin receptors.

## A diagnostic circulating miRNA signature in human gliomas as an orchestrator of tumor invasiveness by targeting mediators of cell invasion: is it useful for miRNA-based complementary therapies?

**Fig. 10:**
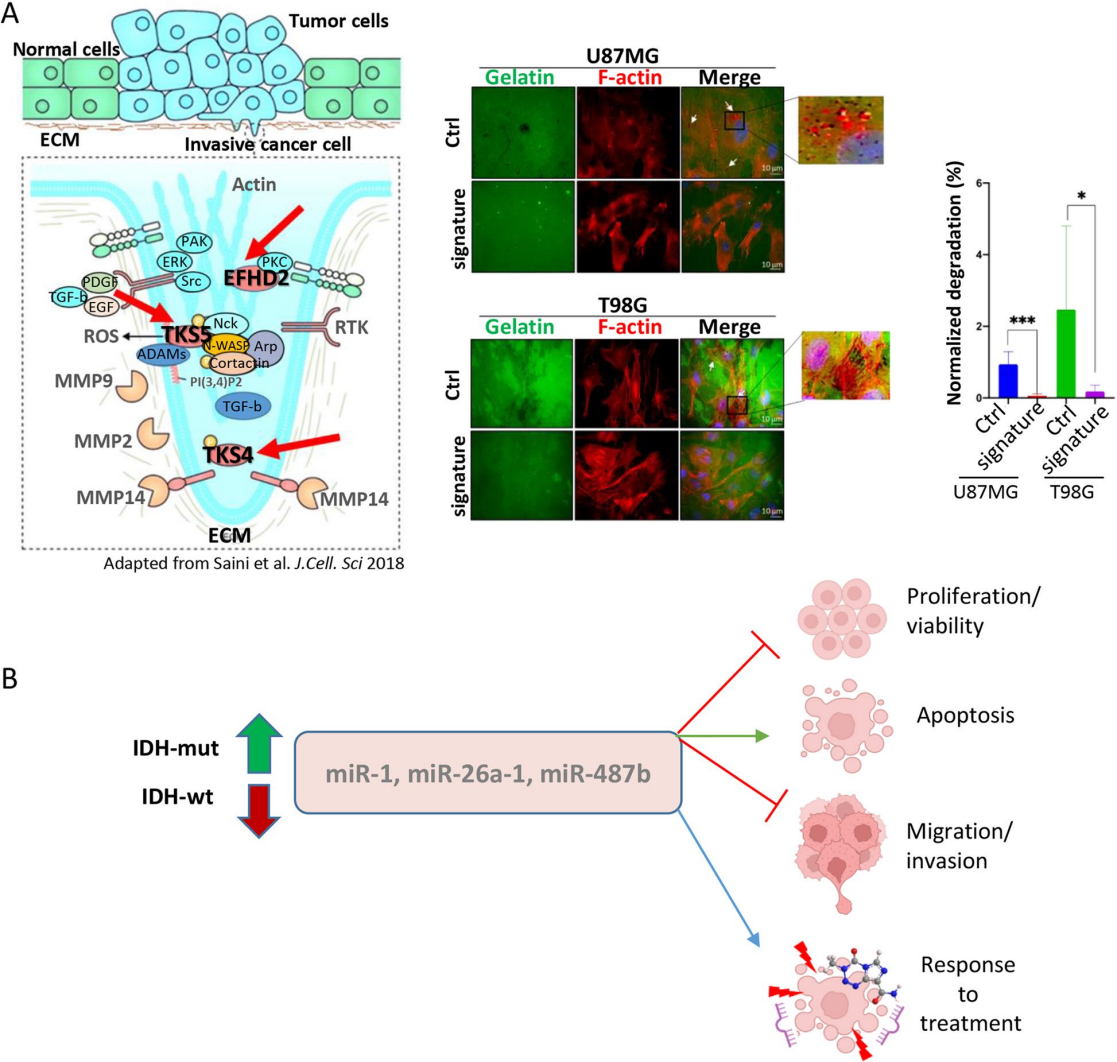
Impact of the miR-1/-26a-1/-487b signature on the main biological functions in glioma. **A** The miRNA signature inhibits invadopodia activity by targeting invadopodia-related genes that encode for TKS4, TKS5 and EFHD2 proteins. **B** Schematic representation of the main three-miRNA signature impacted functions, including response to treatment

*Ana Belén Díaz Méndez* (IRCCS, Regina Elena National Cancer Institute, Rome, IT). Gliomas are diffusely growing brain tumors and challenging cancers for diagnosis and treatment. The identification of genetic/epigenetic markers has led to an integrated histological and molecular diagnosis. Among the key genetic events, the isocitrate dehydrogenase (IDH) mutations are noteworthy from a diagnostic and a prognostic point of view [23]. MiRNAs are important players in glioma by modulating cancer-related processes and have been observed to be altered in both tumor tissues and biofluids. Recently, we identified a serum miRNA signature (miR-1, miR-26a-1 and miR-487b) deregulated in glioma patients according to the IDH mutation profile and correlated with the prognosis. We also showed that the three-miRNA signature displays oncosuppressive functions in glioma cells with the most marked effect on proteins crucial for migration and invasion [24] (Fig. 10A). Of note, we also have, even if preliminary, data indicating that the signature increases the response to treatment of glioma cells, suggesting an effect as a sensitizer to treatments (Fig. 10B). Altogether, our data suggest that the identified signature, having an impact on glioma biology, can pave the way for the development of novel miRNA-based complementary therapies useful for patients’ management.

## Che-1/miR-590-3p/TAZ axis sustains multiple myeloma disease

**Fig. 11:**
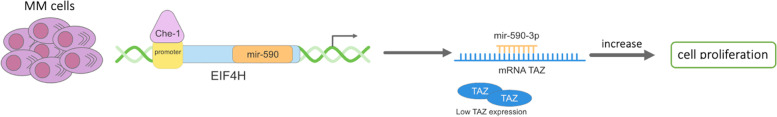
Model of the axis Che-1/miR-590-3p/ TAZ that sustains the tumor progression in MM cells. In MM cells Che-1 is upregulated and recruited on the promoter of EIF4H, the host gene of miR-590-3p. The miRNA downregulates TAZ both on transcriptional and post-translational level promoting cell proliferation

*Maria Chiara Cappelletto* (IRCCS, Regina Elena National Cancer Institute, Rome, IT). Multiple myeloma (MM) is a blood tumor characterized by the malignant accumulation of monoclonal plasma cells in the bone marrow. Among the pathological consequences of the MM, we found defects in osteogenesis characterized by osteolytic lesions, osteopenia, and pathologic fractures. CHE-1/AATF (Che-1) is a co-transcriptional factor that promotes MM proliferation. We show that high Che-1 expression in MM contributes to maintaining low level of WWTR1 (TAZ), a transcriptional coactivator downstream of the Hippo-signaling pathway. We report that miR-590-3p, deriving from the mRNA splicing of the EIF4H host gene, is able to target TAZ, thus contributing to downregulate TAZ expression in MM. We demonstrate by in vivo and in vitro experiments that Che-1 transcriptionally induces miR-590-3p expression by activating EIF4H gene. Moreover, we found that MM cells secrete miR-590-3p in the bone marrow of both patients and a mouse model, providing data that this miRNA inhibits TAZ expression and the physiological transcriptional expression of osteogenic-related genes (Fig. 11). Our findings unveil an unexplored novel Che-1/miR-590-3p/TAZ axis in MM tumorigenesis by providing a rationale to explore its therapeutic potential in metastatic bone lesions [25–27].

## Role of microRNA dependent PTEN downregulation in the resistance to PI3K inhibitor alpelisib in head & neck squamous cell carcinoma

**Fig. 12:**
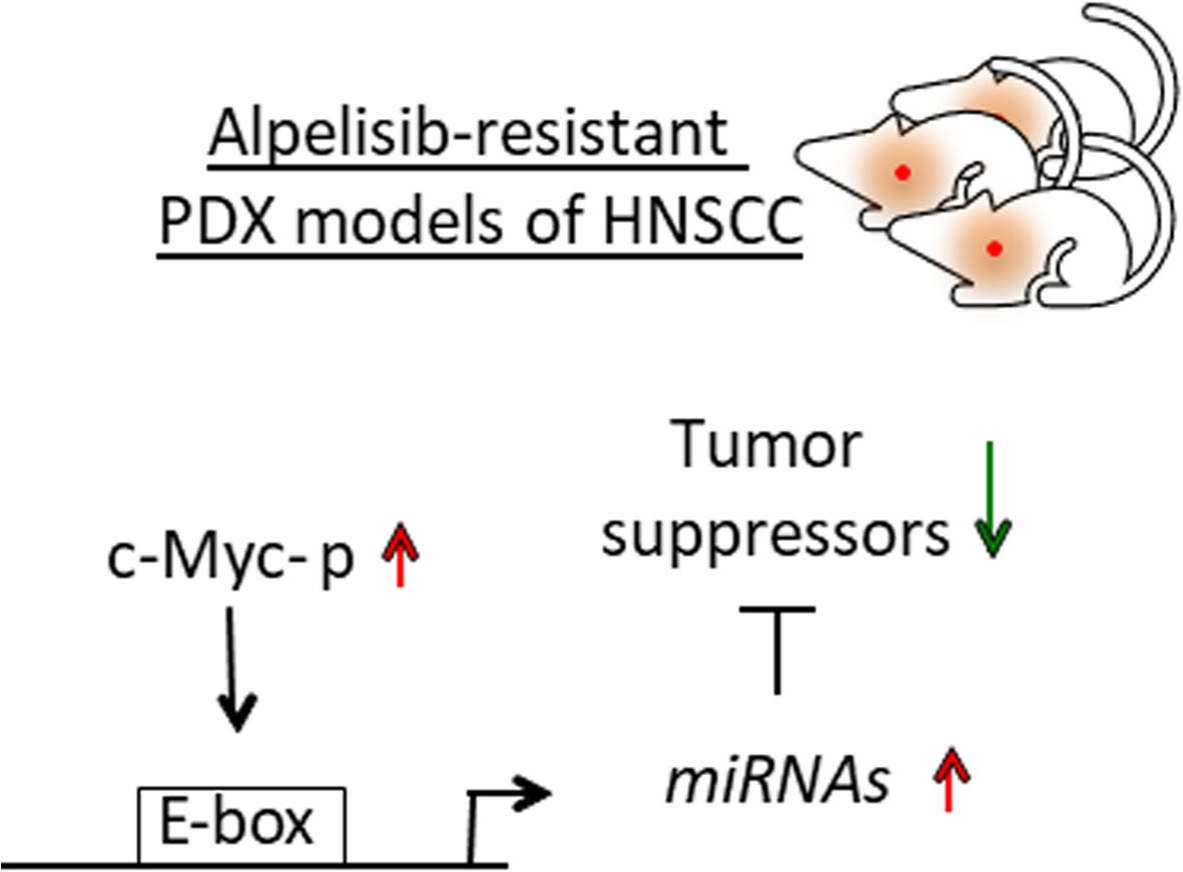
c-Myc-dependent transcription activation of a signature of mRNAs allows the acquisition of resistance to the alpelisib treatment, in patient-derived xenografts of head and neck squamous cell carcinoma

*Claudio Pulito* (IRCCS, Regina Elena National Cancer Institute, Rome, IT). Head and neck squamous cell carcinomas (HNSCCs) represent the sixth most common cancer with the lowest five- year survival rate among the different tumors, mainly due to acquisition of resistance to therapy without acquisition of any further mutations [28]. We revealed post-transcriptional non-genetic events that contribute to the acquisition of resistance to the PI3Kα inhibitor, alpelisib. In particular, we found a signature of 20 microRNAs differently expressed between responder-patient derived xenografts (PDX) and not responder ones to the alpelisib treatment, whose expression correlate with the tumor weight reduction. Of note, 15 of these miRNAs have been found to inhibit translation of several proteins involved in cancer, such as tumor suppressors. Through different biological approaches, we pointed out the transcriptional role of c-Myc as the main driver of the acquisition of resistance and found new molecular targets to overcome the alpelisib-acquired resistance in HNSCC cell lines (Fig. 12).

## The HIPKs’ circular RNA in gastro-Intestinal cancer

**Fig. 13:**
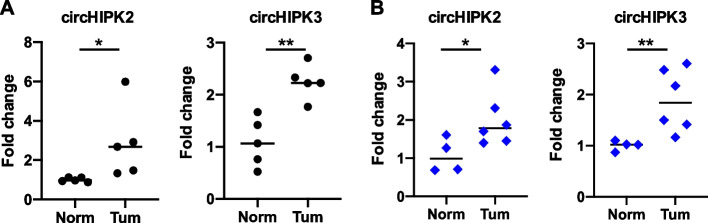
A-B RT-qPCR of circHIPK2 and circHIPK3 in: **A** tumor tissue of CRC patients (Tum) and matched normal tissue (Norm); **B** plasma samples of healthy volunteers (Norm) and CRC patients (Tum)

*Laura Monteonofrio* (IRCCS, Regina Elena National Cancer Institute, Rome, IT). Circular RNAs (circRNAs) are emerging as key regulators of cancer-related biological hallmarks, such as cell proliferation, apoptosis, immune regulation and angiogenesis. CircRNAs are functional RNAs generated by the back-splicing of exons, intron, or intro-exon mRNA regions [29]. An unbiased, broad-spectrum study has shown that the homeodomain interacting protein kinases, HIPK2 and HIPK3, produce abundant circRNAs. Both HIPKs have an evolutionarily conserved kinase domain (KD) coded by exon 2, whose back-splicing yields the HIPK circRNAs (circHIPK2/3) [30]. Among the Gastro-Intestinal cancer, colorectal cancer (CRC), is the second most common cause of cancer death. Our preliminary results show that in CRC patients both circHIPK2 and circHIPK3 are upregulated in tumor tissues compared with their matched normal tissues (Fig. 13A). Moreover, the analysis of the levels of circulating circHIPK2/3 in plasma samples from patients with CRC, shows that both circulating circHIPK2 and circHIPK3 are higher compared to healthy voluntaries (Fig. 13B).

## About motifs, non-coding RNAs and cancer therapy

**Fig. 14:**
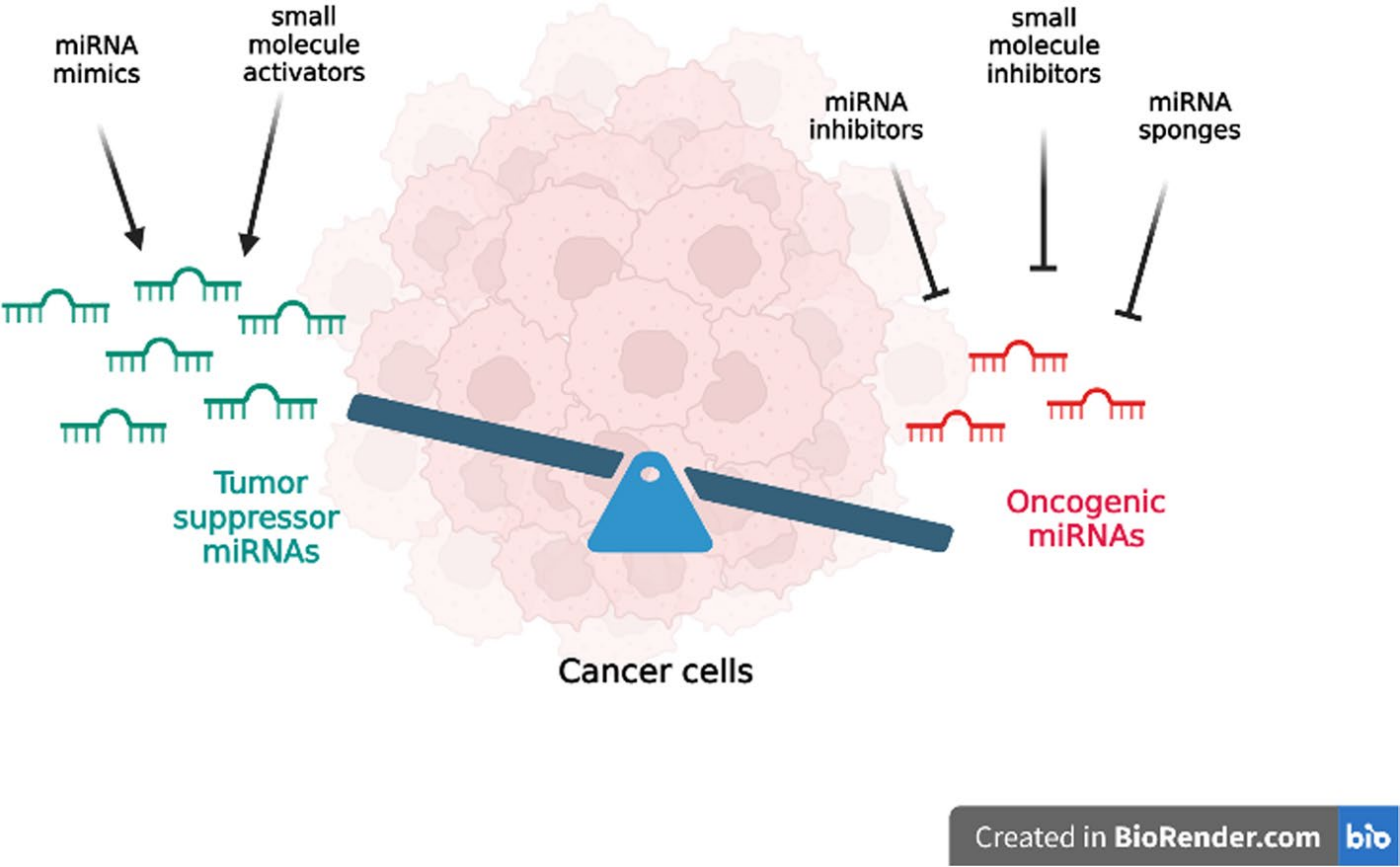
The biological activity of miRNAs involved in cancer and the therapeutic applications

*George A. Calin* (MDAnderson, University of Texas, Houston, USA). Non-coding RNAs (ncRNAs) are a class of RNA molecules that are not translated into proteins. Among the different types of ncRNAs, microRNAs (miRNAs) and long non-coding RNAs (lncRNAs) are the most predominant transcripts. They play a crucial role in regulating protein-coding genes at the levels of transcription, stability, or translation within eukaryotic cells. These distinct subclasses of ncRNAs have been extensively studied in recent years due to their functional diversity and relevance to many biological processes and diseases. These ncRNA transcripts are highly conserved sequences and are widely appreciated as key regulators of multiple cancer hallmarks, including cell proliferation, differentiation, invasion, apoptosis, drug resistance, metastasis and genomic instability. miRNAs and lncRNAs are the most extensively studied ncRNA subclasses. miRNAs, which are typically small endogenous ncRNA molecules, are involved in regulating a wide range of developmental and physiological processes. The two main approaches for miRNA-based interventions are miRNA antagonism (anti-miRNA/antagomir), which involves inhibiting or repressing target miRNAs, and miRNA replacement therapy (miRNA mimic), which restores the expression or function of target miRNAs (Fig. 14). However, the instability of miRNAs and their complex environment has made the delivery of miRNAs a challenging task. As a result, the development of effective methods for delivering miRNAs to specific tissue sites has become a major focus of recent research. Given the complex spatial structures and diverse biological functions of miRNAs in cancer therapy, they may serve as potential targets for small molecules, providing opportunities to modulate key cellular processes for therapeutic purposes. Small molecules can bind to RNA structures and modulate their function, leading to the identification of an increasing number of small-molecule RNA ligands and a growing interest in RNA-based therapeutics. These findings offer promising opportunities for developing new paths for the pharmacological modulation of ncRNAs with small-molecule drugs in cancer therapy. As a result, ncRNA therapeutics and small molecules have entered human clinical trials with encouraging outcomes, including the development of novel engineered natural and synthetic miRNA nanocarriers and in vivo approaches.

## Biomarkers for early detection and screening of lung cancer

**Fig. 15:**
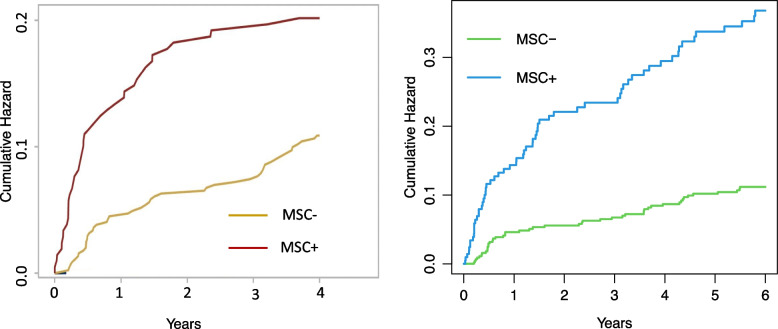
Cumulative lung cancer incidence by baseline vs yearly MSC repetition in participants with an LDCT-indeterminate/positive (LDCT +) finding. (*n* = 655)

*Gabriella Sozzi* (IRCCS, National Cancer Institute, Milan, IT). The most effective healthcare intervention for LC after smoking cessation is early detection by low-dose computed tomography (LDCT) screening. Large randomized trials have demonstrated that LC screening with LDCT reduces LC mortality in heavy smokers. We started already 10 years ago with a strategy of personal lung cancer risk refinement through the evaluation of blood miRNAs and developed a miRNA signature classifier (MSC) able to stratify screening participants according to the risk to develop lung cancer. BioMILD is the first screening trial that prospectively tested the efficacy of a combined LDCT-microRNA approach in 4119 smokers. A positive LDCT and MSC result at baseline predicted individual LC incidence at 5 years, with a major effect of MSC for LDCT-indeterminate/positive individuals (Fig. 15) [31]. These findings establish a basis for the adoption of personalized screening and prevention programs.

## A miRNA blood test for early detection of gastric cancer

*Jit Kong Cheong* (National University of Singapore and Mirxes, Singapore). Circulating miRNAs have been shown to be actively secreted from cells in organs and tissues via extracellular vesicles, such as exosomes, to serve as active messengers for cell-to-cell communication. As compared to circulating tumor DNA, circulating miRNAs are highly abundant and stable in liquid biopsy, and they possess some tissue specificity and functional information as reported in a huge body of literature, and they also have relatively high comprehensive profiling feasibility. Although miRNAs exhibit features of a good disease diagnostics analyte, they are extremely small as compared to a typical messenger RNA and hence it is technically challenging to detect miRNAs. With the advent of new technologies such as the mirxes modified stem-loop mediated reverse transcription quantitative PCR (mSMRT-qPCR) assay chemistry, more than 400 miRNAs can now be detected in as little as 200 μL of serum [32, 33]. Armed with this cutting-edge technology, scientists from MiRXES collaborated with the Singapore Gastric Cancer Consortium to identify and develop GASTROClear, a miRNA blood test for the early detection of gastric cancer [34]. Gastric cancer is the 5^th^ most commonly diagnosed cancer and the 4^th^ deadliest cancer worldwide, with high prevalence in Asia. The GASTROClear test is now a CE-marked and Singapore Health Science Authority-approved Class C in vitro diagnostics (IVD) that is commercially available in Singapore and the ASEAN region, with ongoing registration trials in China and Japan. Using GASTROClear, Project CADENCE (NCT05633342) and the establishment of non-coding RNA (ncRNA) Core Facilities at the National University of Singapore (NUS, Singapore) and the Beth Israel Deaconess Medical Centre (BIDMC, USA) as examples, Dr Cheong emphasized the importance of public–private collaboration to reduce time-to-market duration of practice-changing innovations such as novel miRNA-based IVDs.

## Prediction of cancer treatment response from histopathology images through imputed transcriptomics

**Fig. 16:**
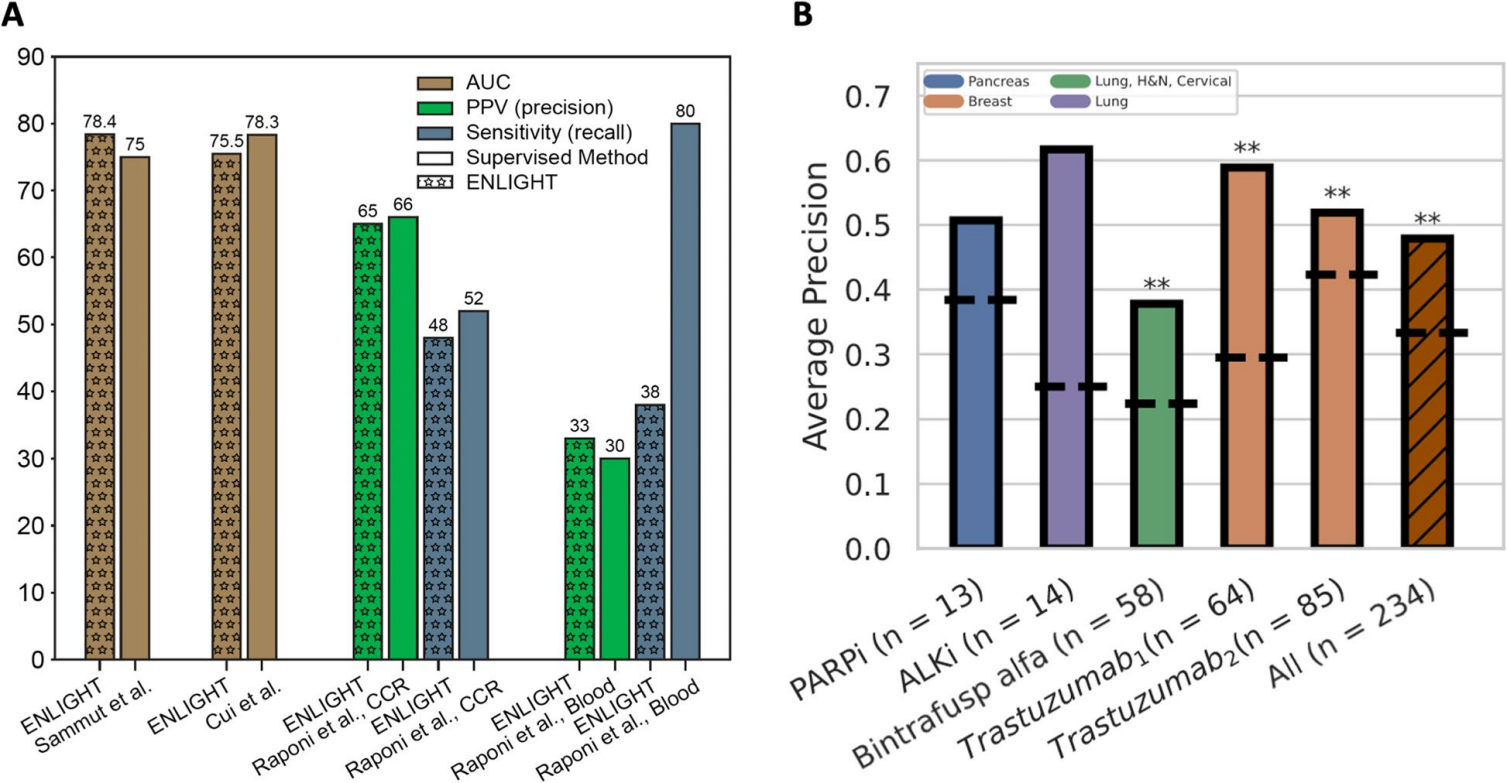
ENLIGHT-matched treatments are associated with better patient response: The Odds Ratio (OR, Y axis) for response of cases matched by ENLIGHT vs. cases unmatched by ENLIGHT for individual blinded cohorts, along with the OR for the aggregation of all cohorts. Drug and sample sizes are given on the X-axis labels. The red horizontal dashed line denotes an OR of 1, as expected by chance. Cohorts for which OR is significantly larger than 1 according to Fisher’s exact test are denoted with asterisks. **a** Based on bulk transcriptomics. **b** Based solely on H&E slides

*Ranit Aharonov* (Pangaea Biomed, Tel Aviv, ISR). Precision oncology (PO) is advancing into mainstream clinical practice, demonstrating significant survival benefits. However, eligibility and response rates remain limited in many cases. We present ENLIGHT [35] a transcriptomics-based computational approach that identifies clinically relevant genetic interactions and uses them to predict responses to a variety of therapies in multiple cancer types, without training on previous treatment response data. We study ENLIGHT as a PO tool, aimed at prioritizing treatments for patients, based on measuring bulk tumor transcription (Fig. 16A). We also developed a deep-learning framework that predicts genome-wide tumor gene expression from H&E slides and created ENLIGHT-DP [36] by applying ENLIGHT to the predicted expression values (Fig. 16B). We validated our approach on 27 blinded clinical trial datasets, showing that it enhances the ability to predict response [35, 36]. The ability to predict response directly from H&E slides opens up promising opportunities for advancing PO in developing countries.

## Data Availability

Not applicable.

## Abbreviations

PI3K: Phosphoinositide 3-kinases
RNAseq: RNA sequencing
WES: Whole exome sequencing
TRF2: Telomeric repeat binding factor 2
LNPs: Lipid nanoparticles
TNBC: Triple negative breast cancer
IHC: Immunohistochemistry
LncRNA: Long non-coding RNA
EBNA2: Epstein-Barr nuclear antigen 2
PD-L1: Programmed death ligand-1
EBF1: Early B-Cell transcrption factor
saRNA: Small activating RNA
CIC: Cancer immunity cycle
TME: Tumor microenvironment
miRNA: MicroRNA
DLBCL: Diffuse large B-cell lymphoma
HG-SOC: High-grade serous ovarian carcinoma
GSDME: Gasdermin E
ET_A_R: Endothelin A receptor
OS: Osteosarcoma
PCA: Principal component analysis
IDH: Isocitrate dehydrogenase
MM: Multiple Myeloma
HNSCCs: Head and neck squamous cell carcinoma
PDX: Patient derived xenograft
circRNA: Circular RNA
KD: Kinase domain
CRC: Colorectal cancer
LDCT: Low-dose computed tomography
MSC: miRNA signature classifier
mSMRT-qPCR: Multiplex reverse transcription quantitative PCR
